# Deep learning in Cobb angle automated measurement on X-rays: a systematic review and meta-analysis

**DOI:** 10.1007/s43390-024-00954-4

**Published:** 2024-09-25

**Authors:** Yuanpeng Zhu, Xiangjie Yin, Zefu Chen, Haoran Zhang, Kexin Xu, Jianguo Zhang, Nan Wu

**Affiliations:** 1 Department of Orthopedic Surgery, Peking Union Medical College Hospital, Peking Union Medical College and Chinese Academy of Medical Sciences, Beijing, 100730 China; 2 Beijing Key Laboratory for Genetic Research of Skeletal Deformity, Beijing, 100730 China; 3 Key Laboratory of Spinal Deformity Research and Application of Big Data, Beijing, 100730 China; 4 State Key Laboratory of Complex Severe and Rare Diseases, Peking Union Medical College Hospital, Beijing, 100730 China

**Keywords:** Scoliosis, Deep learning, X-Rays, Meta-analysis, Cobb angle

## Abstract

**Purpose:**

This study aims to provide an overview of different deep learning algorithms (DLAs), identify the limitations, and summarize potential solutions to improve the performance of DLAs.

**Methods:**

We reviewed eligible studies on DLAs for automated Cobb angle estimation on X-rays and conducted a meta-analysis. A systematic literature search was conducted in six databases up until September 2023. Our meta-analysis included an evaluation of reported circular mean absolute error (CMAE) from the studies, as well as a subgroup analysis of implementation strategies. Risk of bias was assessed using the revised Quality Assessment of Diagnostic Accuracy Studies (QUADAS-2). This study was registered in PROSPERO prior to initiation (CRD42023403057).

**Results:**

We identified 120 articles from our systematic search (*n* = 3022), eventually including 50 studies in the systematic review and 17 studies in the meta-analysis. The overall estimate for CMAE was 2.99 (95% CI 2.61–3.38), with high heterogeneity (94%, *p* < 0.01). Segmentation-based methods showed greater accuracy (*p* < 0.01), with a CMAE of 2.40 (95% CI 1.85–2.95), compared to landmark-based methods, which had a CMAE of 3.31 (95% CI 2.89–3.72).

**Conclusions:**

According to our limited meta-analysis results, DLAs have shown relatively high accuracy for automated Cobb angle measurement. In terms of CMAE, segmentation-based methods may perform better than landmark-based methods. We also summarized potential ways to improve model design in future studies. It is important to follow quality guidelines when reporting on DLAs.

**Supplementary Information:**

The online version contains supplementary material available at 10.1007/s43390-024-00954-4.

## Introduction

**Fig. 1:**
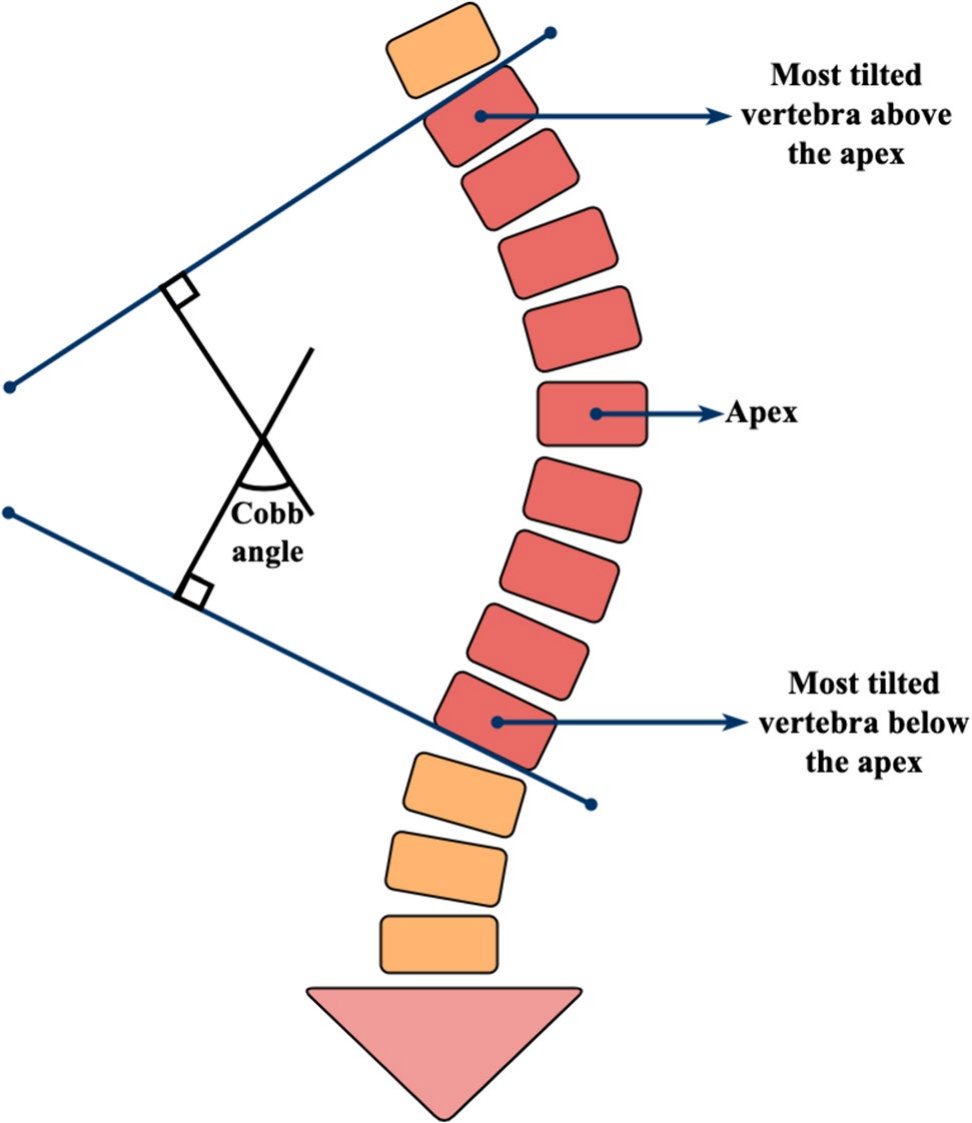
The measurement method of Cobb angle

Scoliosis is a complex spinal deformity that involves both lateral and rotational curvatures of the spine, which can have severe implications for both cardiac and respiratory functions [1, 2]. Adolescent idiopathic scoliosis is the most prevalent type of scoliosis, characterized by a lateral curvature of the spine of greater than or equal to 10° in individuals between 10 and 18 years of age, without vertebral deformity [3, 4]. The Cobb angle, defined as the angle between lines drawn parallel to the endplates of the two vertebrae that exhibit the greatest degree of tilt on X-rays, is the standard for assessing idiopathic scoliosis (Fig. 1) [5]. Accurate measurement of the Cobb angle is crucial for clinicians to design individualized surgical and medicinal interventions, making a dependable method for its measurement of utmost importance.

Currently, the manual measurement of the Cobb angle using anterior–posterior (AP) and lateral (LAT) X-rays remains the primary and gold standard approach for scoliosis assessment [6]. However, this method has several limitations. First, it is time consuming and arduous, as it requires manually counting the vertebrae, a task that can be challenging due to the low image contrast on X-rays, making it difficult to identify vertebral borders and corners. Second, the determination of end vertebrae greatly depends on the clinician’s subjective judgment, which can result in significant inter- and intra-observer errors [7, 8]. Therefore, there is a pressing need to develop both an efficient and a reliable tool that can automatically measure the Cobb angle.

Deep learning algorithms (DLAs), a subset of artificial intelligence, have made significant advances in recent years due to increases in both computational power and the availability of extensive datasets [9]. DLAs have the potential to enable descriptive, predictive, and prescriptive analyses that would be unattainable with manual methods [10, 11]. Recently, several studies have applied DLAs, based on either vertebra segmentation or landmark prediction methods, to automatically and directly estimate Cobb angles from X-rays, with promising results [12, 13].

However, there is a lack of comprehensive reviews and investigations into automated Cobb angle estimation. In this systematic review and meta-analysis, we aimed to evaluate the performance of current DLAs for measuring Cobb angles on X-rays, highlighting the current state of the field, identifying common flaws, and providing evidence-based recommendations for future research.

## Methods

This systematic review and meta-analysis adhered to the guidelines outlined in the Preferred Reporting Items for Systematic Reviews and Meta-Analyses (PRISMA) statement [14], and the study protocol was registered in PROSPERO prior to initiation (CRD42023403057).

### Search strategy

This study examined research papers on the development or validation of DLAs for automated Cobb angle estimation on X-rays. A comprehensive search was conducted across various databases, including PubMed (U.S. National Library of Medicine), Embase (Elsevier), Web of Science (Clarivate), IEEE Xplore (Institute of Electrical and Electronics Engineers), Cochrane, and arXiv (print archive), up until September 2023. The scope of this review was restricted to English-language publications, with no limitations on publication dates. The complete search strings, encompassing keywords and constraints, are detailed in Additional File: eTable1.

### Inclusion and exclusion criteria

The predetermined inclusion criteria were as follows: First, the patients included in these studies must not have undergone surgical intervention and must have vertebral deformities. Second, the performance assessment of the DLAs must be measured through the circular mean absolute error (CMAE) metric, which can either be directly extracted or calculated from the original data provided in the manuscript. Third, study results must be validated using clinical expertise, either internally or externally, by radiologists. Finally, Cobb angles should be measured solely on AP X-rays, as opposed to CT or MRI scans. It is worth noting that we excluded (1) letters, scientific reports, and narrative reviews; (2) non-deep learning models; and (3) studies that were based on animal or non-human samples, or that presented non-original data. To ensure consistency in the selection process, two researchers screened the titles and abstracts of the retrieved articles, subsequently applying the inclusion and exclusion criteria.

### Data extraction

Literature accepted for analysis was reviewed by two researchers, ZYP and CZF, using the PRISMA guidelines. After discussing and resolving disagreements, any remaining issues were resolved with the participation of a third researcher, ZHR. Information collected from studies included: (a) year of publication, (b) first author, (c) country of the first author, (d) title of the article, (e) study design, (f) name or the base architecture of the deep learning (DL) model, (g) size of training set, (h) whether there was an internal/external validation or test cohort and its size, (i) the source of the dataset, (j) whether the model was based on multi-models, (k) study algorithms’ design based on segmentation or landmark strategy, (l) whether the model was based on one or two stage models, (m) the CMAE score and its standard deviation (SD) (if more than one score was investigated, we chose the highest score and the SD could be reported or recalculated), (n) symmetric mean absolute percentage (SMAPE) and its SD.

### Quality assessment

Quality assessments were performed independently by two investigators. Their evaluation was based on the revised Quality Assessment of Diagnostic Accuracy Studies (QUADAS-2) [15]. In case of any disagreement, a third researcher, ZHR, was consulted to resolve the issue through discussion. This quality control tool comprises four components: patient selection, index testing, reference standard, and flow and timing. The final criterion is based on the risk of bias, with particular attention given to concerns about applicability. The risks of bias were rated as high, low, or unclear.

### Statistical analysis

The CMAE is commonly used as the evaluation metric for a regression algorithm, and is adapted in most studies for Cobb angle estimation [12]. It is defined as:

$$CMAE = \frac{1}{N}\mathop \sum \limits_{K = 1}^{N} \left| {gt_{k}^{a} - pre_{k}^{a} } \right|$$

representing the absolute error between ${gt}_{k}^{a}$ and ${pre}_{k}^{a}$, which are the Cobb angle calculated by manual and DLAs, respectively.

Studies to be included in the meta-analysis needed to have reported the outcome of interest (i.e., CMAE score), in combination with an SD, standard error (SE), and/or a 95% confidence interval (95% CI). The SD was statistically assessed from the SE and/or the 95% CI. Meta-analysis was performed on aggregated data from all studies that met the inclusion criteria. We used the R software (version 4.3.0) for meta-analysis. When estimating the overall effect size of the current DLAs, we used a random effects model. Statistical tests were considered significant when *p* < 0.01.

The Higgins I^2^ test was used to examine and quantify inconsistency among the included studies. Values above 75% indicate high heterogeneity between groups, while values between 25 and 50% suggest moderate heterogeneity. Values below 25% indicate no significant heterogeneity between groups. We also conducted subgroup analyses based on the algorithm strategy (segmentation or landmark methods). Sensitivity analysis was performed by sequentially removing each study, and publication bias was tested using a funnel plot and the Egger’s publication bias test.

## Results

### Search results

**Fig. 2:**
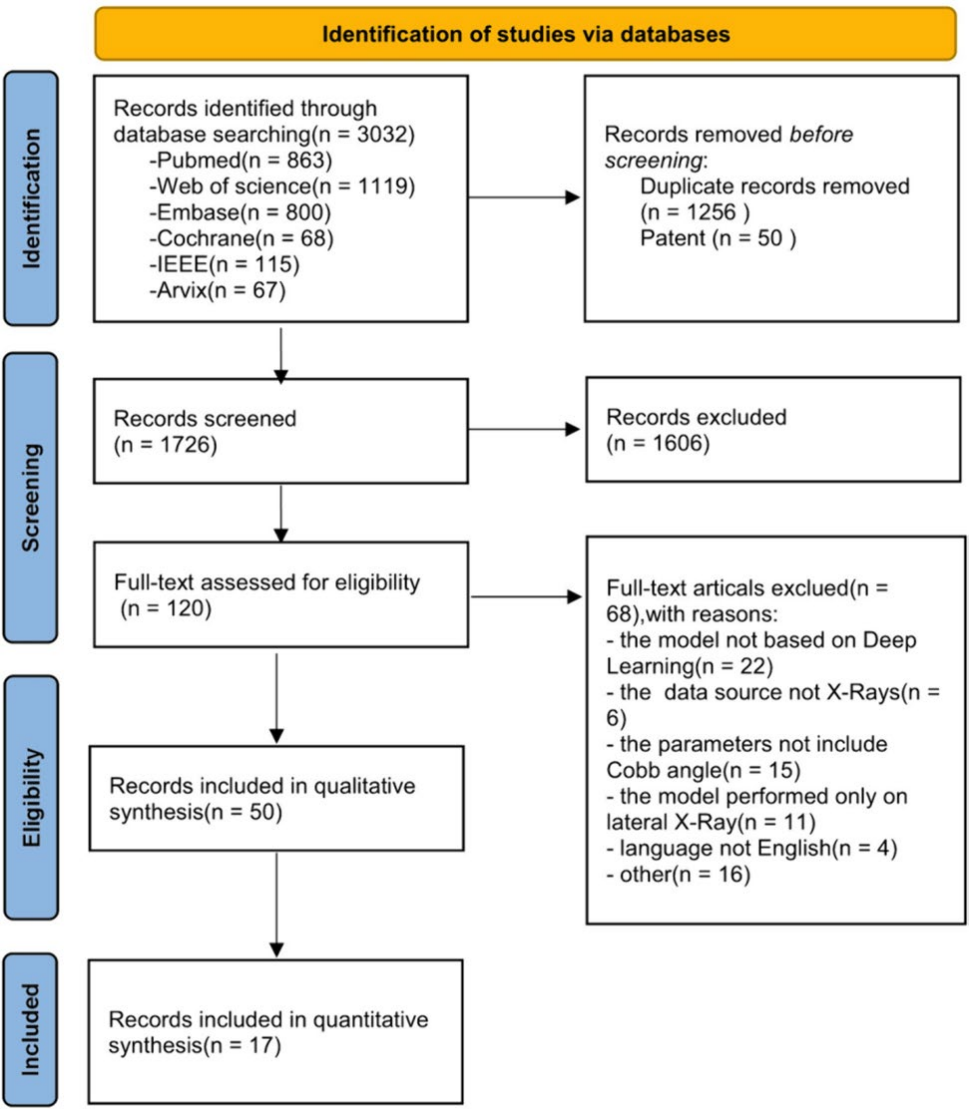
PRISMA flowchart of systematic literature search

Our initial systematic search of various databases yielded a total of 3032 studies. Following the removal of duplicates and patents, 1726 articles were subjected to title and abstract assessment. Among these, 120 articles were eligible for inclusion, and subsequently, 50 studies were included in the systematic review. Of these, 17 studies met the eligibility criteria for the meta-analysis, as they employed evaluation metrics such as CMAE and contained adequate quantitative data for the performance of the models. Figure 2 depicts the flowchart of the selection process for the included studies.

### Review of the included studies

A total of 55 deep learning models were identified by the 50 included studies published between 2016 and 2023, with the majority of the models (35 out of 55) originating from China. The details of the included models can be found in Additional File: eTable2. Remarkably, only three studies were conducted as prospective research, and only one was conducted as a multicenter study. The models used or the base model architectures presented included various types of convolutional neural networks (CNNs), such as U-net[16], ResNet[17], Deeplab V3 + [18], and HRNet[19], among others. Several studies described the participant demographics of the training datasets [17, 20–23]. Furthermore, the MICCAI Challenge on Accurate Automated Spinal Curvature Estimation (AASCE) 2019 was organized to investigate semi-automatic and automatic spinal curvature estimation algorithms, providing a standard evaluation framework with a set of X-rays [24]. The dataset of the challenge included a total of 707 spinal AP X-rays, with 609 in the training set and 98 in the testing set (https://aasce19.grand-challenge.org). As the challenge dataset was open, 21 of the 55 models were trained and tested on this public dataset [24–39]. Among the remaining models, 24 were used on local and private datasets [16–18, 20–22, 40–48], 5 on both local and public datasets [49–53], 2 on other public datasets [18, 54], and 3 were not mentioned in the studies [55–57].

In terms of model design strategies, we categorized them into two groups, as did in previous studies [12, 13, 31]: Landmark-based methods typically involve directly identifying the four corners of each vertebra to establish landmarks and subsequently computing the Cobb angles based on these landmarks. Segmentation-based methods, on the other hand, involve segmenting all the vertebrae using specialized models, followed by the application of machine learning algorithms to detect vertebral corners and calculate the Cobb angles. Out of the total number of models considered, 30 were based on landmark estimation, 22 on segmentation, and 3 incorporated both methods.

The key consideration of this meta-analysis was to determine the evaluation metric, and the performance of these models was evaluated using two main metrics: CMAE and SMAPE. Of the 35 models evaluated based on CMAE, the values ranged from 1.07° to 17.13°. Among these, 17 models were reported with standard deviations, making them suitable for meta-analysis, and detailed information about the selected studies is summarized in Table 1. For the 28 models that reported SMAPE, the values ranged from 7.32 to 45.99, though only 4 models included standard deviation measures. Ultimately, after careful consideration, CMAE was chosen as the metric for meta-analysis.

**Table 1:** The characteristics of the included studies included in meta-analysis

Year-Author	Country	Architecture or Name of the model	Size of training set	Size of test set	The source of the dataset	The strategy of model design	CMAE ± SD
2016-Aubert et al. [21]	Canada	DNN + SSM	425	121	Local hospital	Landmark based	4.20 ± 3.80
2019-Tu et al. [49]	China	DU-Net	600	200	Local and public datasets	Landmark based	2.92 ± 1.55
2018-Tan et al. [16]	China	U-Net	510	47	Local hospital	Segmentation based	1.70 ± 1.20
2019-Horng et al. [43]	China	CNN	NR	35	Local hospital	Segmentation based	2.85 ± 1.86
2019-Zhang et al. [45]	China	CNN	840	120	Local hospital	Landmark based	3.29 ± 1.48
2020-Imran et al. [46]	USA	U-Net	80	15	Local hospital	Segmentation based	2.41 ± 1.57
2020-Kim et al. [50]	South Korea	CNN	431	128	Local and public dataset	Landmark based	3.51 ± 3.89
2020-Fu et al. [20]	China	LCE-Net	720	240	Local hospital	Segmentation based	3.15 ± 3.09
2022-Caesarendra et al. [51]	Brunei	CNN	481	70	AASCE challenge and local datasets	Landmark based	2.14 ± 1.43
2022-Huang et al. [17]	China	ResNet101	200	300	Local hospital	Landmark based	2.92 ± 1.75
2022-R. Maaliw et al. [48]	Philippines	Residual U-Net	180	20	Local hospital	Segmentation based	1.57 ± 1.04
2022-Sigurdson et al. [22]	Canada	CNN	382	100	Local hospital	Segmentation based	2.80 ± 2.80
2021-Sun et al. [23]	China	CenterNet	145	36	Local hospital	Landmark based	2.20 ± 2.00
2018-Wu et al. [12]	Canada	MVC-Net	526	526	Local hospital	Landmark based	4.04 ± 0.99
2022-Zhang et al. [13]	China	MPF-net	2738	274	Local hospital	Landmark based	3.52 ± 1.07
2022-Yao et al. [31]	China	W-Transformer	609	98	AASCE challenge	Landmark based	3.76 ± 4.31
2020-Zhang et al. [19]	China	HRNet	367	73	Local hospital	Landmark based	4.15 ± 3.11

*CNN* convolutional neural network, *DNN* deep neural network, *SSM* statistical shape model, *LCE-Net* Landmark and Cobb angle estimation network, *MVC-Net* Multi-View Correlation Network, *MPF-Net* Multi-task, Proposal correlation, Feature fusion Network, *HRNet* high resolution network, *AASCE challenge* Accurate Automated Spinal Curvature Estimation challenge, *SD* standard deviation, *CMAE* circular mean absolute error, *USA* United States of America, *NR* not reported

### Risk of bias

**Fig. 3:**
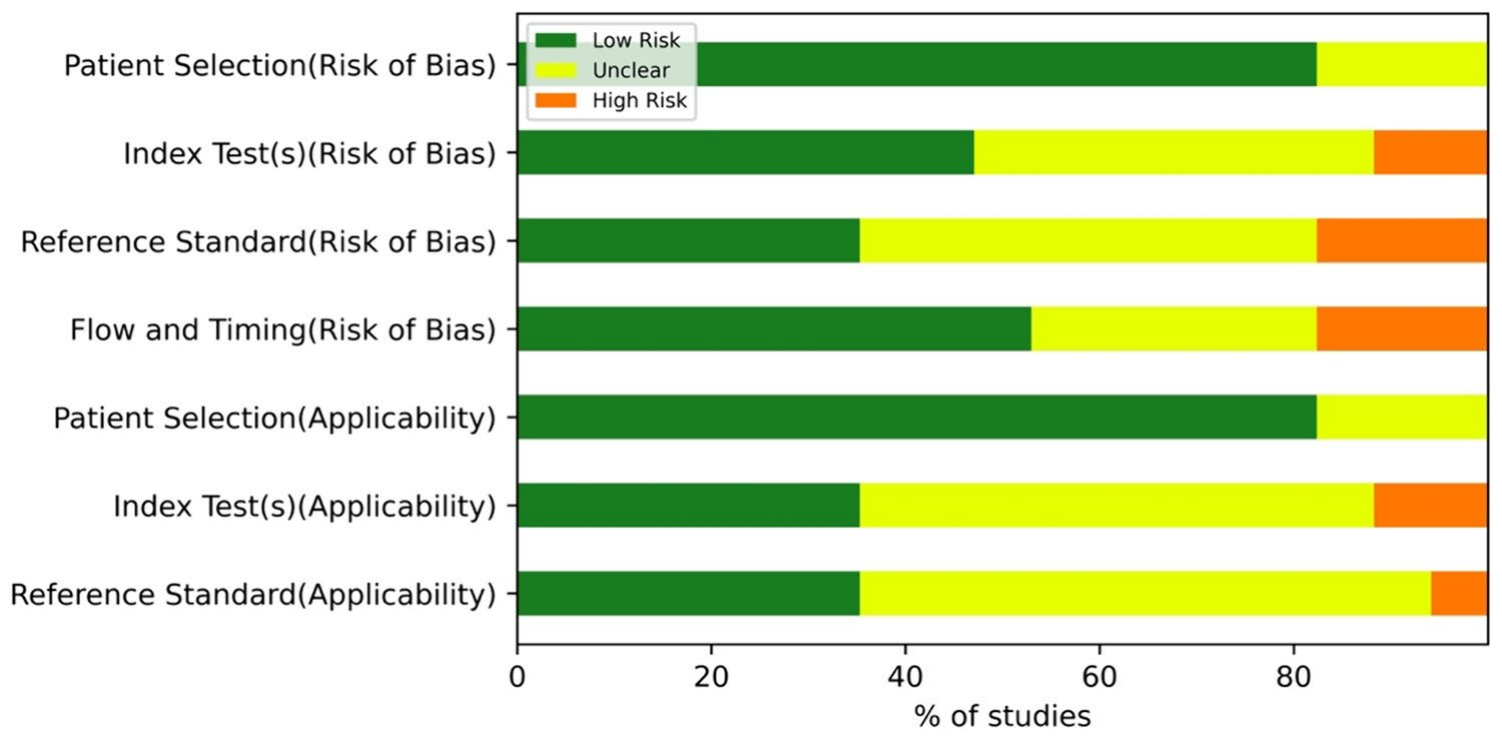
QUADAS-2 summary plots. Risk of bias and applicability concerns summary about each QUADAS-2 domain presented as percentages across the 17 included studies

We also evaluated the quality of the studies as well as the risk of bias using the revised QUADAS-2 tool (Fig. 3 and Additional File: eTable3). In the “patient selection (risk of bias)” domain, all studies were considered to be at relatively low risk (82.35%) or unclear risk (17.65%) of bias. In the “index test (risk of bias)” domain, eight studies (47.06%) were at low risk of bias, and seven (47.18%) were unclear. In “reference standard (risk of bias)”, six studies (35.29%) were at low risk of bias, and eight (47.06%) were unclear. In addition, in terms of “flow and timing (risk of bias)”, nine studies (52.94%) were scored with a low risk of bias, and five (29.41%) were unclear.

### Meta-analysis of the included studies

**Fig. 4:**
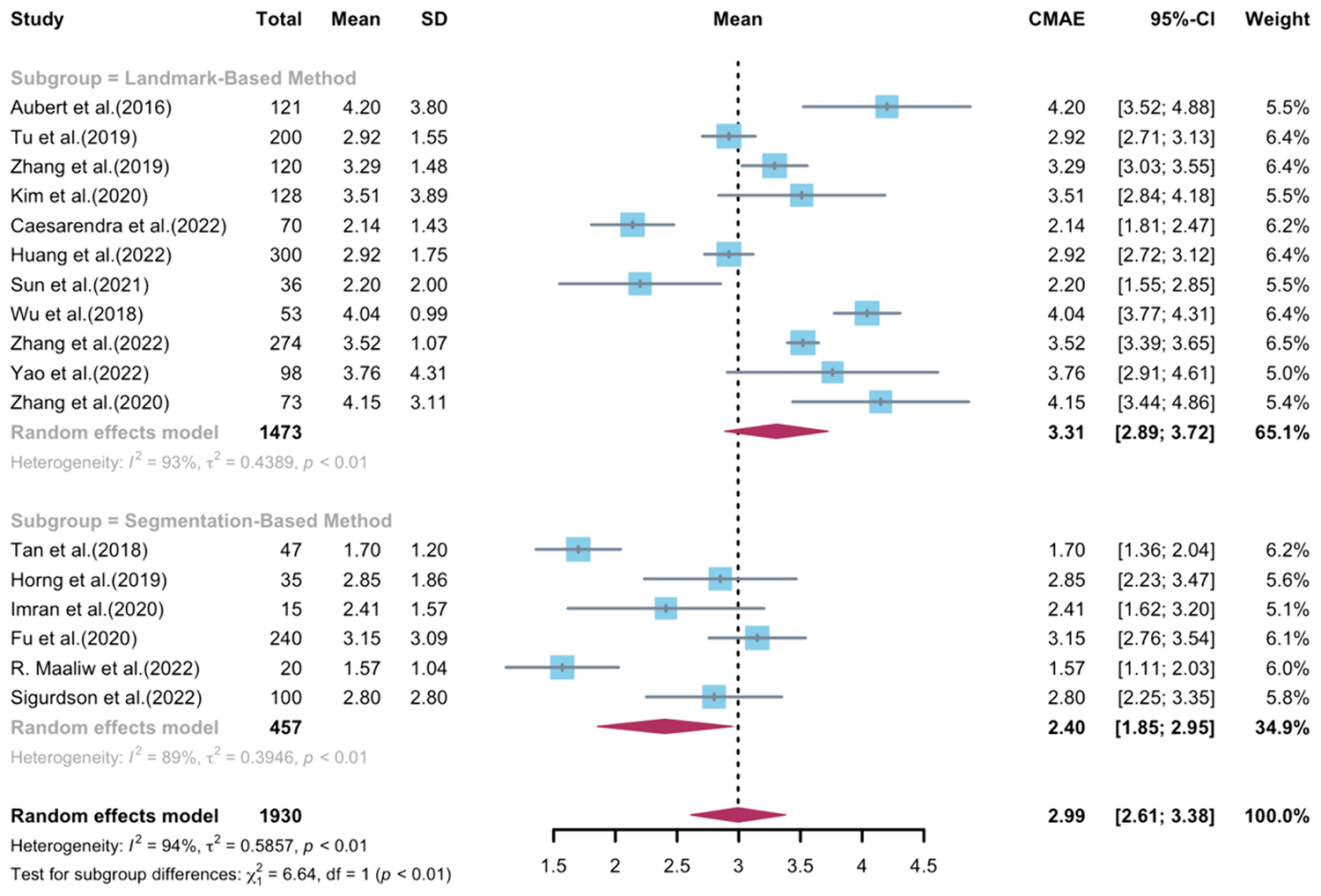
Forest plot of the included studies that assessed the automated measurement of Cobb angle (random effects model). *CI* confidence interval, *CMAE* circular mean absolute error, *SD* standard deviation

The meta-analysis involved 17 models that were described in 17 individual studies [12, 13, 16, 17, 19–23, 31, 43, 45, 46, 48–51]. The overall CMAE estimate was 2.99 (95% CI 2.61–3.38), indicating a relatively small measurement error **(**Fig. 4**)**. However, the high degree of heterogeneity observed among the studies (94%, *p* < 0.01) suggested that the models differed significantly in their accuracy. Sensitivity analysis was conducted by sequentially removing each study, and the results indicated consistent findings among all studies (Additional File: eFigure1).

For the subgroup analysis of implementation strategies, 11 models using landmark-based estimation methods were included [12, 13, 17, 19, 21, 23, 31, 45, 49–51], with a corresponding CMAE estimate of 3.31 (95% CI 2.89–3.72) and a heterogeneity estimate of 93% (*p* < 0.01) **(**Fig. 4**)**. On the other hand, another subgroup comprised 6 models based on segmentation methods [16, 20, 22, 43, 46, 48], yielding an overall CMAE estimate of 2.40 (95% CI 1.85–2.95) and a heterogeneity estimate of 89% (*p* < 0.01) **(**Fig. 4**)**. Thus, both subgroup meta-analyses revealed high levels of heterogeneity, implying that the models varied widely in their performance. The results of the test for subgroup differences (*p* < 0.01) indicated that the segmentation-based strategy showed a trend toward better accuracy in Cobb angle measurement automation compared to landmark-based methods in terms of CMAE score.

### Publication bias

The studies included in the publication bias analysis were the 17 studies. The funnel plot showed an asymmetrical shape, suggesting potential publication bias among them (Additional File: eFigure2). However, Egger’s publication bias test showed that *P* = 0.283 > 0.05, implying no publication bias in the included studies (Additional File: eFigure3).

## Discussion

Our review illustrates that DLAs can achieve exceptional accuracy in automated Cobb angle estimation on X-rays. The overall CMAE estimate of the included models in this meta-analysis was 2.99 (95% CI 2.61–3.38), which is considered acceptable by spine specialists in clinical practice [58]. The results showed a significant difference in accuracy between these two strategies, suggesting that segmentation-based methods may be more accurate than landmark-based methods. It is important to highlight that although automated Cobb angle estimation is rapidly evolving, it has yet to gain widespread acceptance in routine clinical practice.

There are numerous factors that contribute to errors in automated Cobb angle measurement, including variation in researchers and tools. Gstoettner et al. demonstrated that digital radiography does not necessarily improve measurement accuracy compared to assessment based on printed radiographs, and that the selection and definition of end vertebrae are the main sources of error in Cobb angle measurement [7]. Methods that use algorithmic settings to select the proximal and distal end vertebrae may potentially reduce errors. Traditional machine learning methods, such as support vector machines [59–61], have been used to address this problem before the widespread adoption of deep learning. However, these approaches have limitations, particularly in terms of limited data analysis capability and model performance. Deep learning, on the other hand, has emerged as a promising and powerful approach to automated Cobb angle estimation. Remarkably, the AASCE 2019 significantly boosted the application of DLAs in the accurate automated quantitative estimation of spinal curvature, and many state-of-the-art (SOTA) models have been proposed in this challenge.

Concerning the implementation strategy of these models, we conducted a subgroup meta-analysis. We gained insight into the differences between these strategies: (1) Segmentation subgroup models predominantly used U-net-based models as the baseline for the segmentation part. After segmenting the vertebrae, the minimum bounding rectangle (MBR) algorithm was typically used to obtain the corners of the vertebra, after which the corresponding Cobb angle could be calculated based on these corners. However, due to the MBR algorithm, if a vertebra is not a perfect rectangular shape, even with good segmentation performance, the four vertices obtained by the MBR algorithm may significantly differ from the actual corners of the vertebra. Furthermore, if the adjacent vertebrae are not completely segmented, it is likely that the MBR algorithm will regard the adjoining vertebrae as one, causing problems for subsequent procedures. (2) Landmark subgroup models are generally considered a more direct approach, and this task is similar to the pose-estimation task, which is fundamental in computer science and for which many state-of-the-art (SOTA) models have been proposed, such as HRNet [19]. It is worth noting that current algorithms struggle to identify landmarks when images feature blurry spine boundaries and significant deformations.

We also summarized the development directions that might improve the performance of the models: (1) A two-stage model consists of two DL models, which can split a complex problem into smaller and simpler problems. The process typically involves training a detection network to identify the bounding box of each vertebra and then cropping the vertebra from the image. Finally, each vertebra will be input into another landmark detection model to determine the corners of the vertebra. Remarkably, a two-stage model might be more accurate than a one-stage model, but it also incurs greater time and computational costs [23, 49, 50, 52]. (2) The combination of segmentation and landmark methods might provide an effective approach to improve performance and reduce the Cobb angle error [33, 62]. (3) It is challenging to utilize the information from the patient’s AP and LAT X-rays simultaneously; some studies have designed multi-view convolution layers or modules to incorporate joint features of two-view X-rays, which allows the network to mitigate the occlusion problem by utilizing the structural dependencies of the two views [12, 13, 63].

In clinical practice, segmentation-based methods are generally preferred due to their higher accuracy compared to landmark-based methods. However, this does not imply that every segmentation-based method will consistently outperform landmark-based methods. Beyond the accuracy of specific algorithms, attention must also be given to the model’s parameter count, robustness, and operational efficiency, which are critical for deployment in clinical settings. Integrating the model into existing imaging systems or simplified measurement tools would significantly enhance its practicality.

However, there are still some limitations that need to be addressed in future research. Initially, most studies did not consider the accuracy and rationality of end vertebra selection, which could impact surgical decision-making; instead, they merely compared the angle errors between various methods. Additionally, unlike common tasks such as medical image segmentation or classification, there is no recognized and standardized evaluation framework for Cobb measurement, complicating the comparison of models and the inclusion of all relevant studies in this meta-analysis. Moreover, variations in study design are expected in meta-analyses, reflecting the diverse aims and methodologies pursued by different authors. This diversity among enrolled studies can introduce hidden inconsistencies that affect the final conclusions and their clinical applicability. Furthermore, current studies lack large-scale research verification.

## Conclusions

Results of this systematic review and meta-analysis indicate that automated Cobb angle measurement using DLAs has shown relatively accurate results. Regarding the CMAE, our results suggest that segmentation-based methods might outperform landmark-based methods. However, there remains room for improvement in terms of standardization and precision to facilitate broader adoption in routine clinical practice.

## Supplementary Information

Below is the link to the electronic supplementary material.

Supplementary file1 (DOCX 422 KB)

## Data Availability

All data generated or analyzed during the study are included in the published paper.
