# Supplementary material for: Deep learning in Cobb angle automated measurement on X-rays: a systematic review and meta-analysis
Source: Spine Deform. 2024 Sep 25;13(1):19–27. doi: 10.1007/s43390-024-00954-4 (PMC11729091; doi:10.1007/s43390-024-00954-4)
Supplement: Supplementary file 1 — Supplementary file1 (DOCX 422 KB) [file 43390_2024_954_MOESM1_ESM.docx]

## eTable1: Search strategy used in each database.

| **Database** | **Search Strategy** | **Results** |
| --- | --- | --- |
| **PubMed** | (((((‘deep learning’) OR (‘neural network’)) OR (‘artificial intelligence’)) OR (‘machine learning’)) OR (‘algorithm’)) AND (((‘Cobb angle’) OR (‘spinal curve’)) OR (‘spine alignment’)) | **863** |
| **Embase/Web of Science** | (((((TS = (deep learning)) OR TS = (neural network)) OR TS = (artificial intelligence)) OR TS = (machine learning)) OR TS = (algorithm)) AND ((TS = (Cobb angle) OR TS = (spinal curve)) OR TS = (spine alignment)) | **800/1119** |
| **Cochrane** | (("All Metadata": deep learning) OR ("All Metadata": neural network) OR ("All Metadata": artificial intelligence) OR ("All Metadata": machine learning) OR ("All Metadata": algorithm)) AND (("All Metadata": Cobb angle) OR ("All Metadata": spinal curve) OR ("All Metadata": spine alignment)) | **68** |
| **IEEE-Xplore** | (((((‘deep learning’) OR (‘neural network’)) OR (‘artificial intelligence’)) OR (‘machine learning’)) OR (‘algorithm’)) AND (((‘Cobb angle’) OR (‘spinal curve’)) OR (‘spine alignment’)) | **115** |
| **arXiv** | order: -announced_date_first; size: 50; include_cross_list: True; terms: AND all=Cobb angle; OR all=spinal curve; OR all=spine alignment | **67** |

*Note: It should be noted that the majority of the literatures found on arXiv is related to computer science. In order to include as many literatures as possible and due to the limited search results on arXiv, we have adopted a search strategy that only includes keywords related to curves to expand the search scope.*

**

**

**
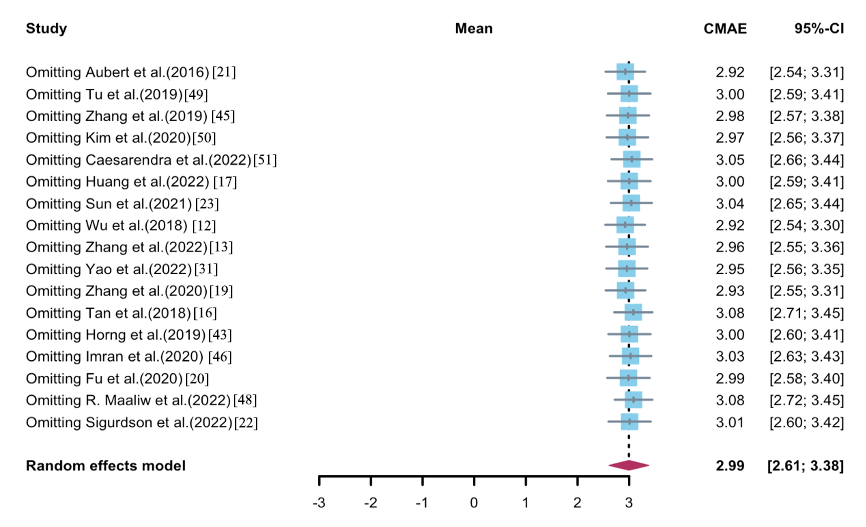
**

**eFigure1****: Sensitivity analysis by sequentially removing each study. CMAE, circular mean absolute error.**

**
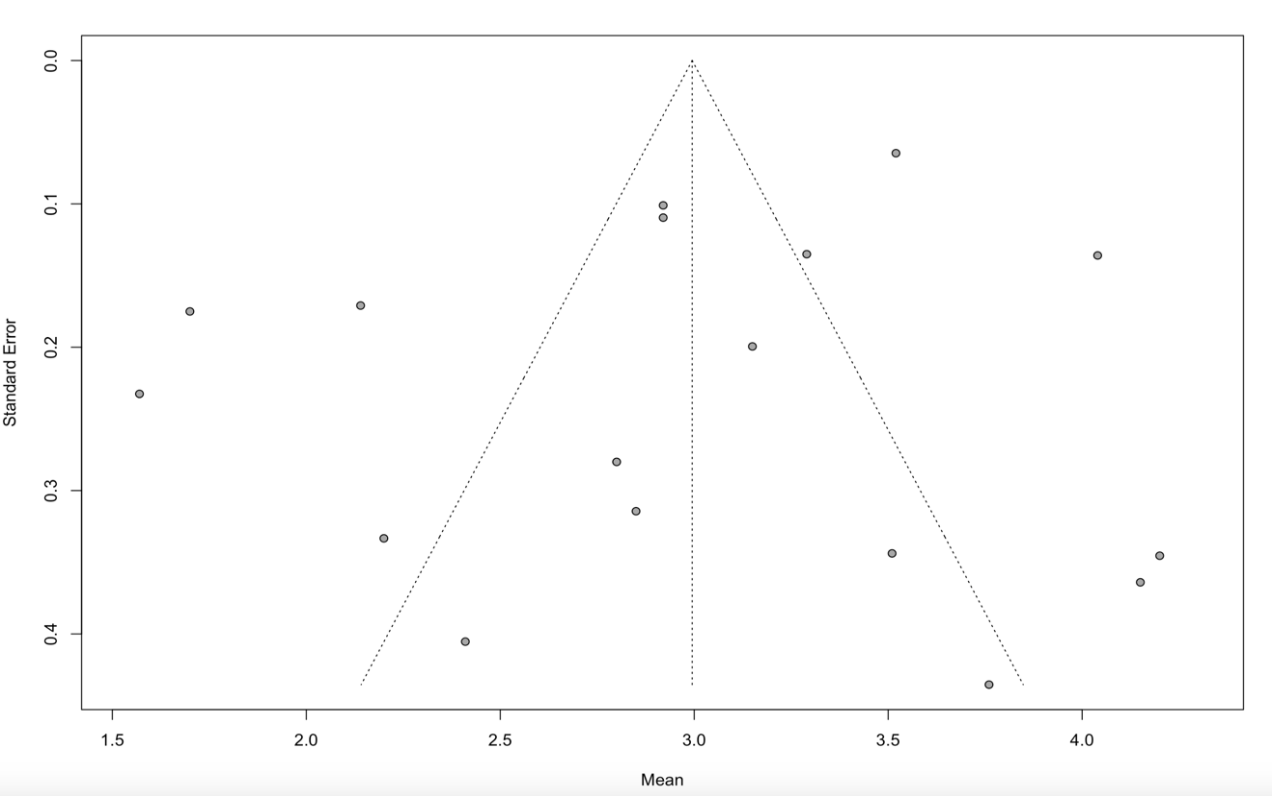
**

**eFigure2: Funnel plot of the included studies.**

**
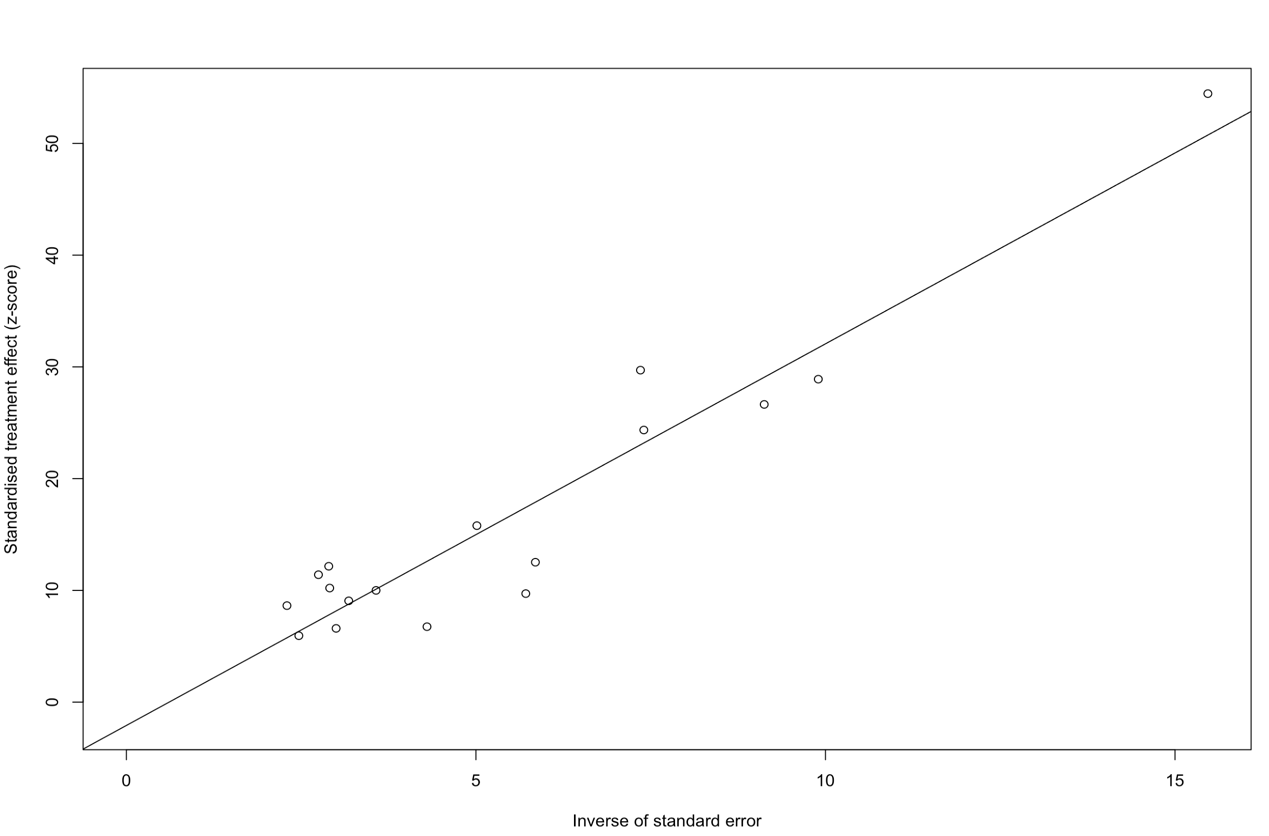
**

**eFigure3: Egger’s publication bias plot.**
